# Frequency-dependent circuits anchored in the dorsal and ventral left anterior insula

**DOI:** 10.1038/s41598-020-73192-z

**Published:** 2020-10-05

**Authors:** Yifeng Wang, Qijun Zou, Yujia Ao, Yang Liu, Yujie Ouyang, Xinqi Wang, Bharat Biswal, Qian Cui, Huafu Chen

**Affiliations:** 1 Institute of Brain and Psychological Sciences, Sichuan Normal University, No. 5, Jing’an Road, Chengdu, 610066 China; 2 The Clinical Hospital of Chengdu Brain Science Institute, MOE Key Lab for Neuroinformation, School of Life Science and Technology, University of Electronic Science and Technology of China, No. 2006, Xiyuan Ave, Chengdu, 611731 China; 3 Department of Biomedical Engineering, New Jersey Institute of Technology, 607 Fenster Hall,University Height, Newark, NJ 07102 USA; 4 School of Public Affairs and Administration, University of Electronic Science and Technology of China, Chengdu, 611731 China

**Keywords:** Psychology, Neural circuits

## Abstract

The hub role of the right anterior insula (AI) has been emphasized in cognitive neurosciences and been demonstrated to be frequency-dependently organized. However, the functional organization of left AI (LAI) has not been systematically investigated. Here we used 100 unrelated datasets from the Human Connectome Project to study the frequency-dependent organization of LAI along slow 6 to slow 1 bands. The broadband functional connectivity of LAI was similar to previous findings. In slow 6-slow 3 bands, both dorsal and ventral seeds in LAI were correlated to the salience network (SN) and language network (LN) and anti-correlated to the default mode network (DMN). However, these seeds were only correlated to the LAI in slow 2-slow 1 bands. These findings indicate that broadband and narrow band functional connections reflect different functional organizations of the LAI. Furthermore, the dorsal seed had a stronger connection with the LN and anti-correlation with DMN while the ventral seed had a stronger connection within the SN in slow 6-slow 3 bands. In slow 2-slow 1 bands, both seeds had stronger connections with themselves. These observations indicate distinctive functional organizations for the two parts of LAI. Significant frequency effect and frequency by seed interaction were also found, suggesting different frequency characteristics of these two seeds. The functional integration and functional segregation of LDAI and LVAI were further supported by their cognitive associations. The frequency- and seed-dependent functional organizations of LAI may enlighten future clinical and cognitive investigations.

## Introduction

The anterior insula is a hub of the salience network (SN), which plays complex roles in most categories of cognition^1^. Several studies have reported the important function of the right anterior insula (RAI) in a variety of attention-demanding tasks^2–4^ and in modulating the default mode network (DMN) and cognitive control network^5,6^. Recently, we have demonstrated the frequency-dependent functional organization of the RAI, supporting its unique role in attention-demanding tasks and inter-network orchestration^7^. Due to the lateralization of AI^8,9^, the left anterior insula (LAI) has rarely been reported in attention-demanding tasks but has been shown in performing emotion-related tasks such as emotional working memory and decision-making^3,10,11^. These studies argue that the LAI has different functional organizations from the RAI which is crucial to its cognitive functions.

Similar to the RAI, the LAI has been divided into dorsal and ventral parts (LDAI and LVAI) using functional magnetic resonance imaging (fMRI) signal^12–14^. The LDAI and LVAI have discriminative functions^1^ and FCs^12^. Although broadband FCs of AI in its entirety show hemispheric lateralization^8^, FCs of the LDAI and LVAI are similar to those of the RDAI and RVAI^12,14^. This is incompatible with distinctive functions of the LAI and RAI^8,9^. Considering the frequency-dependent functional organization of RAI^7^, it is necessary to differentiate subparts of the LAI in different narrow bands for probing its functional organization.

Frequency-specific neural activities have been emphasized in electroencephalographic studies and have recently drawn much attention in fMRI studies^15–17^. The narrow fMRI frequency bands overlap with multiple frequency bands defined previously by electrophysiological studies: slow 4, slow 3, and slow 2^18^. Frequency-dependent characteristics of local brain activity and inter-regional relationship have been widely demonstrated in the low frequency range (< 1 Hz)^19–22^, indicating the functional specificity of narrow low frequency bands. Furthermore, frequency-specific brain activities could enlighten the neural mechanisms of neurological and mental diseases^23–25^. Recently, we demonstrated that the functional segregation of RDAI and RVAI in slow 6-slow 3 frequency bands fits well with externally- and internally-oriented processes, highlighting the frequency-dependent functional organization of the RAI^7^. These findings motivated us to ask, as the symmetrical hubs of the SN, whether and how the functional organizations of LAI are frequency-dependent?

In the present study, we investigated the frequency-dependent FCs of LDAI and LVAI along slow 6 to slow 1 frequency bands using 100 unrelated datasets obtained from the Human Connectome Project (HCP). Considering that the broadband FCs of the dorsal and ventral parts of AI are similar between the left side and right side^12,14^, we hypothesized that different functional organizations between LAI and RAI may exist in narrow frequency bands. Results showed that both LDAI and LVAI are connected with the SN, language network (LN) and anti-correlated with the DMN in slow 6-slow 3 frequency bands, differentiating them from the FC patterns of RAI. Significant connections were limited to the LAI in slow 2 and slow 1 bands, similar to the inner-connections of RAI. Significant frequency effect and frequency by seed interaction were shown in the aforementioned three networks. Furthermore, the cognitive associations for the FC maps of LDAI and LVAI were similar but with different strengths. These findings suggest a unique functional organization of the LAI from the RAI and the important role of band-limited method in revealing the functional organization of LAI.

## Results

### The replication of broadband functional connections of LDAI and LVAI

**Figure 1:**
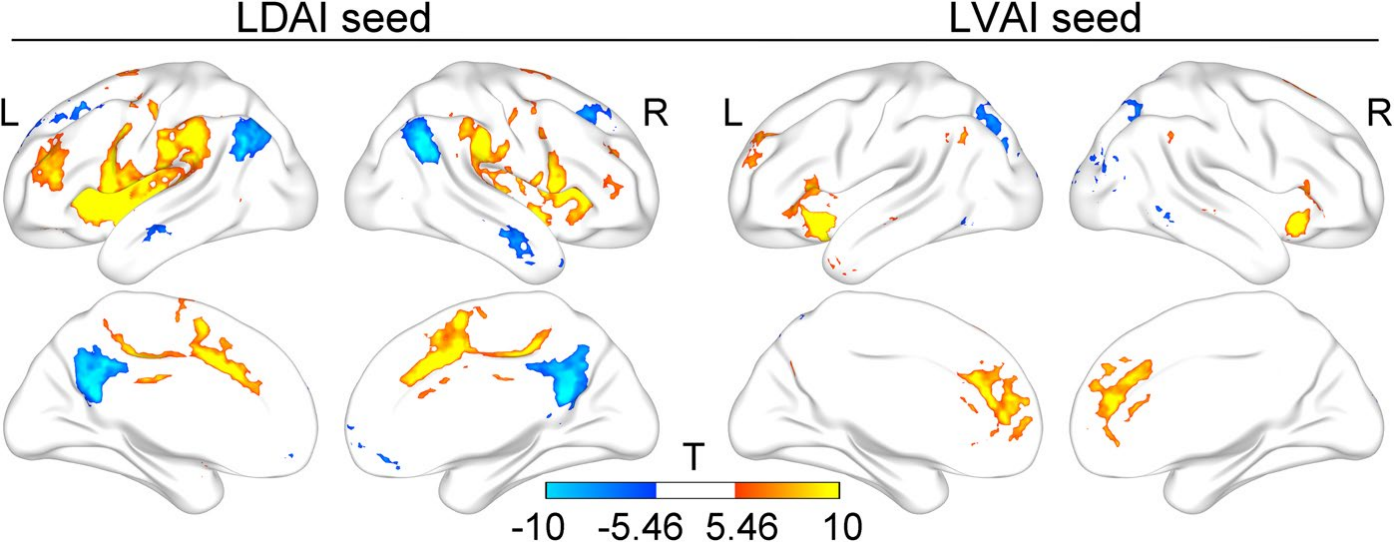
Broadband functional connectivity patterns of the LDAI and LVAI. The positive correlations are similar to those in Deen et al.’s paper^12^ while the anti-correlations are shown between the LDAI and DMN and between the LVAI and lateral parietal-temporal-occipital regions. L: Left; R: Right. Results are visualized with BrainNet Viewer (https://www.nitrc.org/projects/bnv/)^81^.

We first replicated a previous study about the broadband FCs of LDAI and LVAI^12^. As shown in Fig. 1, the LDAI was connected to the dorsal anterior cingulate cortex (dACC), middle cingulate cortex, supplementary motor area, most areas of the insula, and the LN. The Cohen’s d of these regions ranged from 0.61 to 3.99. The LVAI was correlated to the dorsal and rostral ACC, bilateral AI, medial frontal cortex, and inferior parietal lobe. The Cohen’s d of these regions ranged from 0.61 to 5.40. These results are similar to those observed by Deen and colleagues^12^, indicating the repeatability of broadband FC in the LAI. Further, we showed that the anti-correlation between the LDAI and DMN, and between the LVAI and superior parietal lobe, lateral occipital and temporal areas, indicating different functional organizations for the LDAI and LVAI, respectively.

### Effects of seed, frequency, and their interaction

**Figure 2:**
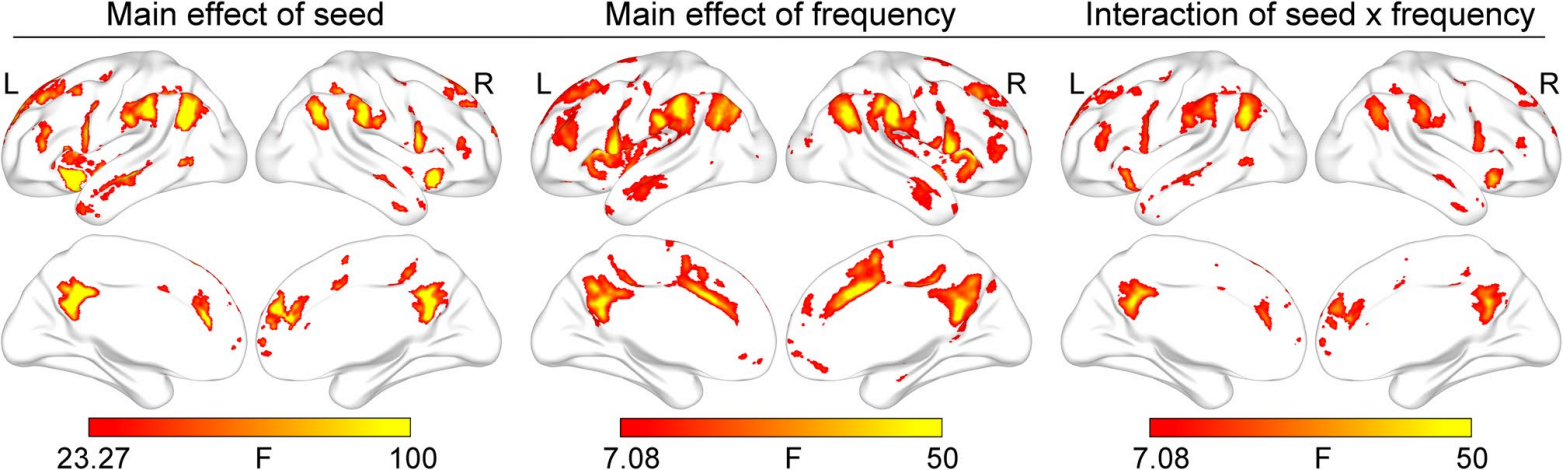
The effects of seed and frequency and their interaction. All effects are primarily located in the salience network, language network, and default mode network. Results are corrected with FWE method (p < 0.05).

Figure 2 shows very similar patterns between the main effect of seed, the main effect of frequency, and the interaction of seed by frequency. All effect regions were located in the SN (e.g., the AI and dorsal anterior cingulate cortex), LN (e.g., the supramarginal gyrus, precentral and postcentral gyri, anterior and opercular part of the inferior frontal gyrus)^26^, and DMN (e.g., the posterior cingulate cortex/precuneus, angular gyrus, medial frontal cortex, lateral temporal cortex, dorsal lateral prefrontal cortex). The Cohen’s f of these regions ranged from 0.15 to 1.32. Of note, the lateral temporal cortex has been classified as one part of the LN or DMN^26,27^. Considering the similar FC patterns between the lateral temporal cortex and regions belonging to the DMN in the present study, we defined the lateral temporal cortex as a part of the DMN. These results mean that both seed and frequency and their interplay are responsible for FC variations of the LAI.

**Figure 3:**
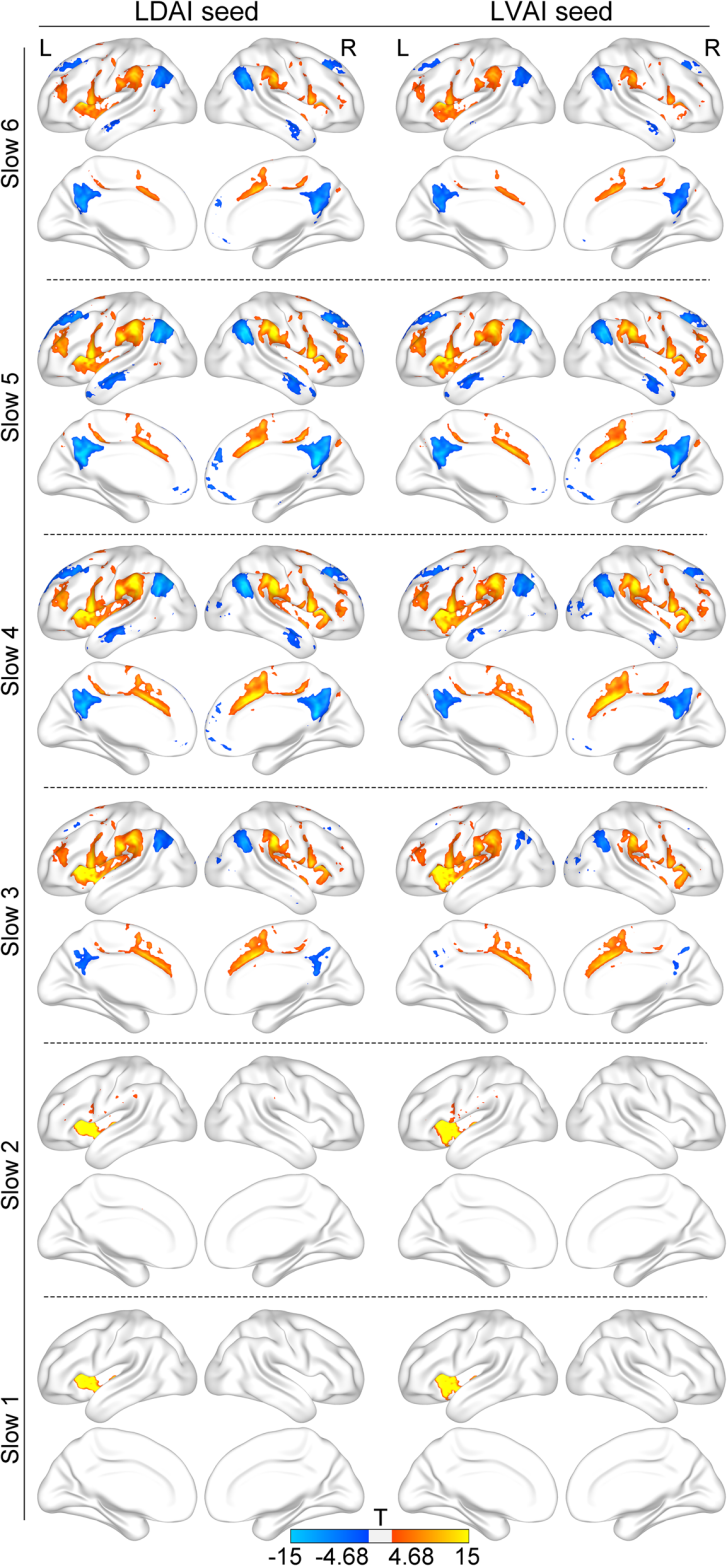
Band-limited functional connectivity patterns of the LDAI and LVAI. Both seeds are positively connected to the salience network and language network, and are negatively connected to the default mode network within slow 6-slow 3 frequency bands. In slow 2 and slow 1 frequency bands, both seeds are positively connected to the left anterior insula itself.

A finer inspection of the FC map of LDAI seed and LVAI seed at each frequency band revealed very similar FC patterns for these two seeds (see Fig. 3). Specifically, both LDAI and LVAI were connected to the SN and LN but anti-correlated to the DMN within slow 6-slow 3 frequency bands. Another FC pattern was found in slow 2 and slow 1 frequency bands with strong connections limited in the LAI. The Cohen’s d of these regions ranged from 0.15 to 1.23.

### Different FC patterns between LDAI and LVAI

**Figure 4:**
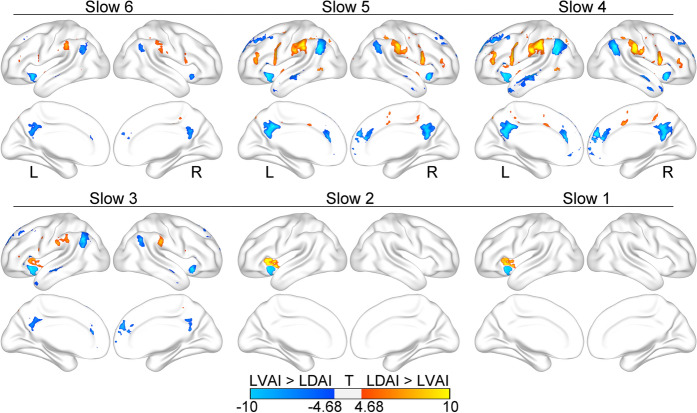
Different functional connectivity patterns of the LDAI and LVAI at each frequency band. The LDAI has a stronger correlation with the language network and anti-correlation with the default mode network whereas the LVAI has a stronger correlation within the salience network at slow 6-slow 3 frequency bands. Both seeds have stronger connections with themselves at slow 2-slow 1 frequency bands.

Within slow 6-slow 3 frequency bands, the LN and DMN had stronger correlation and anti-correlation with the LDAI whereas the SN had a stronger correlation with the LVAI. Within slow 2 and slow 1 frequency bands, both seeds had stronger connections with themselves (see Fig. 4). The Cohen’s d of these regions ranged from 0.15 to 0.41. These findings suggest that LDAI and LVAI have dissociated FC patterns in each frequency band, indicating the functional segregation between them.

### Cognitions associated with frequency-specific FC maps

**Figure 5:**
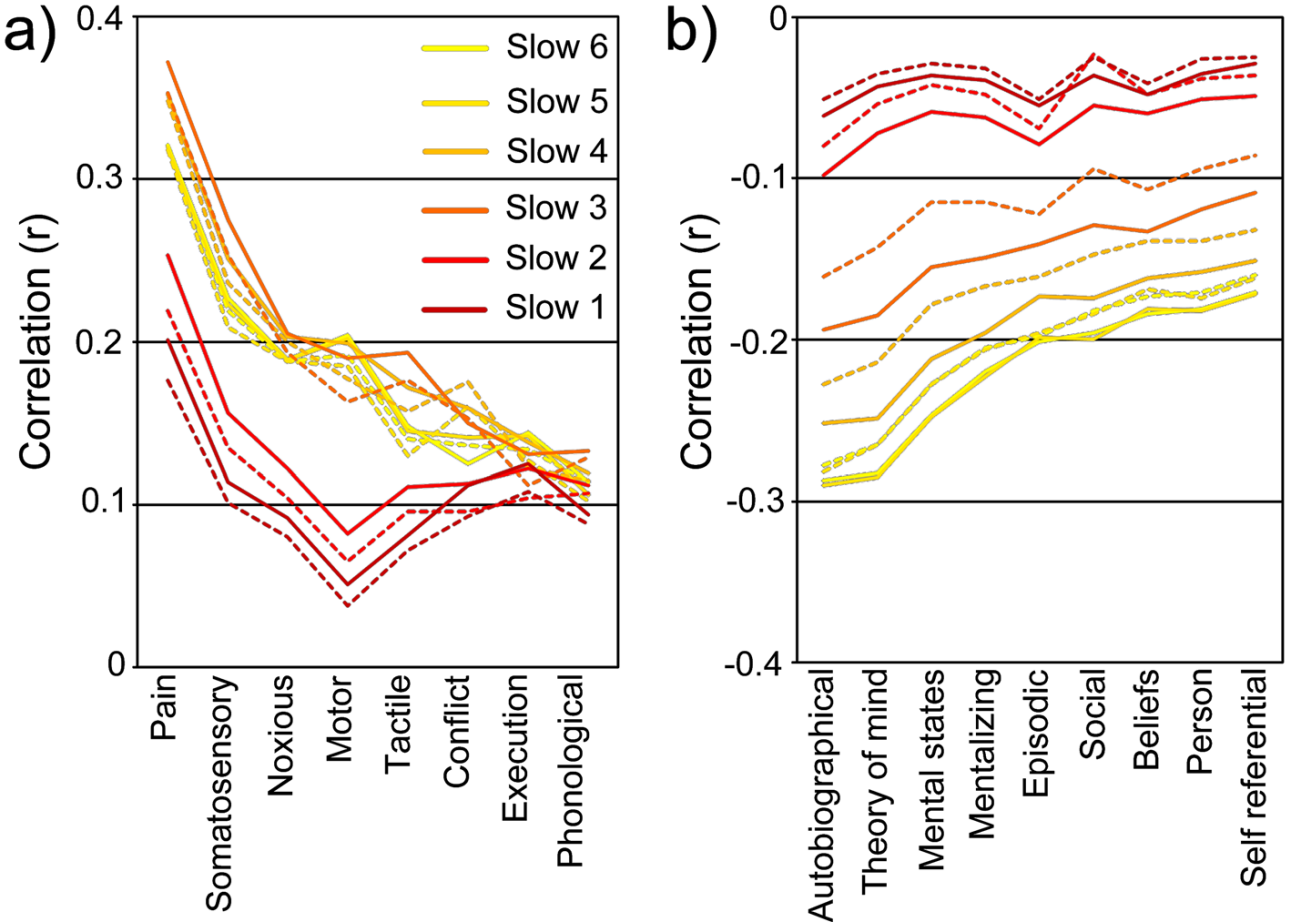
Psychological processes associated with frequency-specific FC maps. The terms were divided into two parts with positive correlation (**a**) and negative correlation (**b**). Solid lines represent the correlation with LDAI while dash lines represent the correlation with LVAI.

Considering the dissociated FC patterns of LDAI and LVAI, we further investigated whether discrepancies are present in the potential psychological functions among frequency-specific FC maps associated with the LDAI and LVAI. This analysis yielded 17 terms of high relevance to frequency-specific FC maps (see Fig. 5). The LDAI and LVAI exhibited dissociated cognitive functional profiles. For positive correlations, compared with the LVAI, the LDAI had stronger correlations with sensory, motor, language, and executive functions but weaker correlation with the conflict function. The relative correlations with conflict were reversed between higher and lower frequency bands. For negative correlations, the LDAI had stronger correlations with all mental functions. These functions are all associated with the DMN. Different correlation patterns with similar cognitions indicate both functional integration and functional segregation of the LDAI and LVAI. These results also support the frequency-specific role of LAI in emotion-related tasks as well as decision-making.

### Changed FC patterns along frequency bands

**Figure 6:**
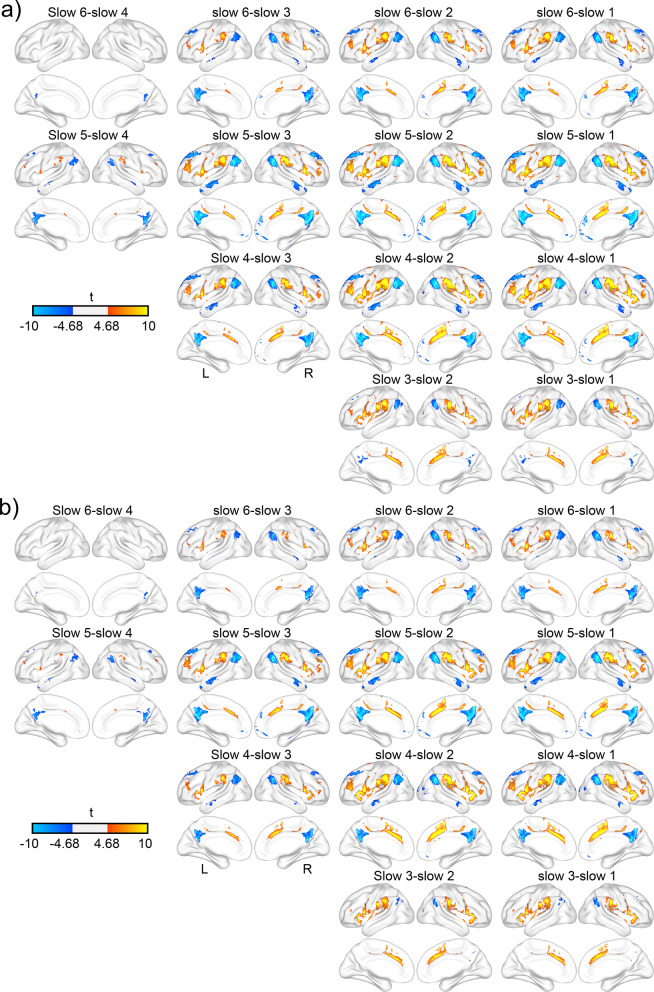
Frequency effect on the functional connectivity of LDAI and LVAI. Panel (**a**) shows frequency difference maps for LDAI while panel (**b**) shows frequency difference maps for LVAI.

As shown in Fig. 6, significant frequency effects appeared widely among frequency bands. Correlations between the LAI and SN and between the LAI and LN as well as anti-correlation between the LAI and DMN tended to weaken as frequency increases. The Cohen’s d of these regions ranged from 0.15 to 0.82. There were no significant differences between slow 6 and slow 5, and between slow 2 and slow 1 frequency bands for the strength of FC, indicating the same FC patterns between these frequency bands.

### Seed by frequency interaction on FC

**Figure 7:**
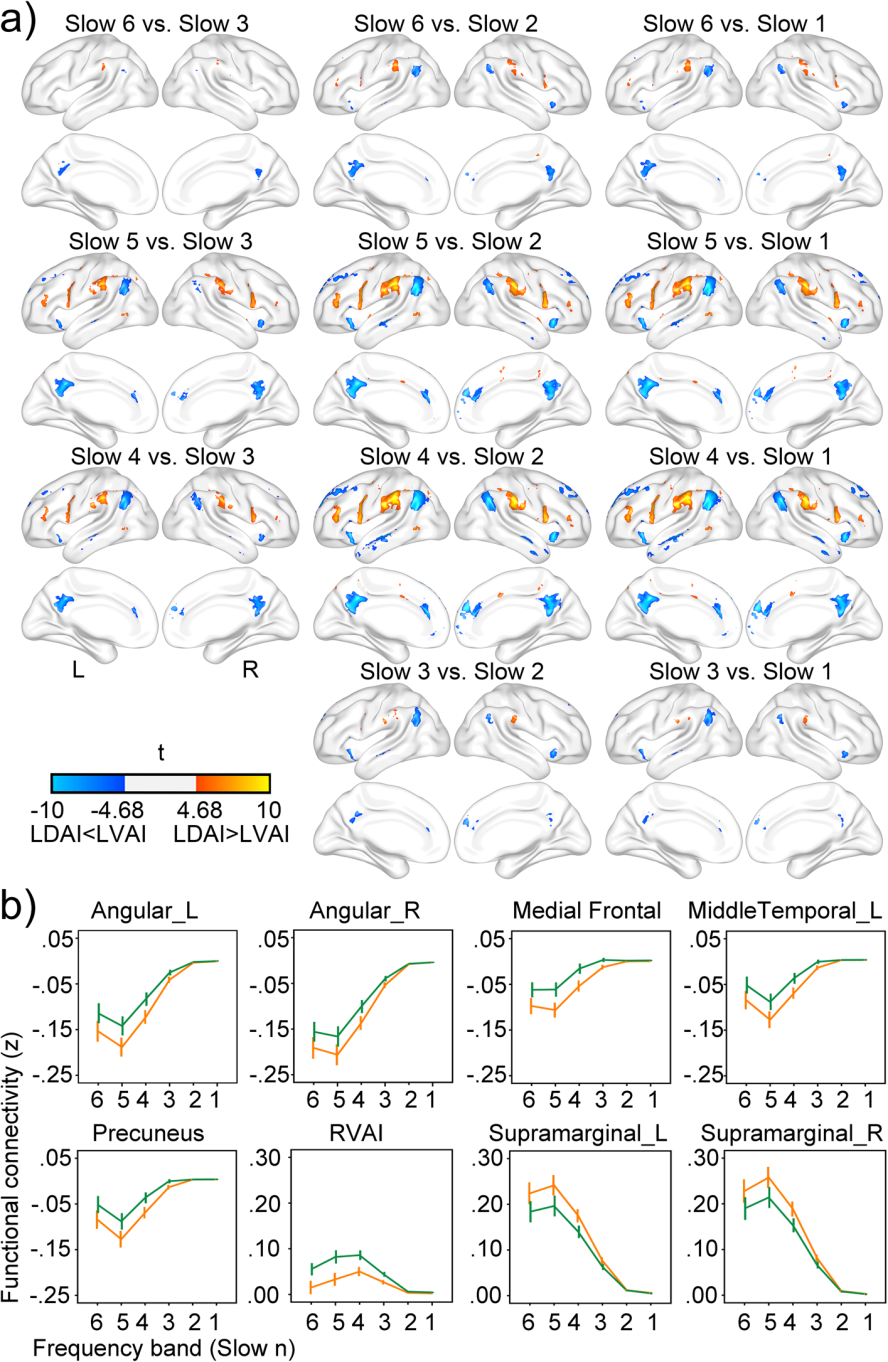
Seed by frequency interaction. Panel (**a**) shows regions with significant seed by frequency interaction. Regions with warm color have more positive correlation with the LDAI whereas regions with cool color have more positive correlation with the LVAI. Panel (**b**) shows the functional connectivity strength in eight representative regions across frequency bands. Orange lines show FCs of the LDAI while green lines show FCs of the LVAI.

Figure 7 shows the details of seed by frequency interaction. Significant interaction appeared in all three networks of SN, LN, and DMN with the Cohen’s d of these regions ranging from 0.15 to 0.37 (see Fig. 7a), suggesting that FC strengths for LDAI seed and LVAI seed attenuate at different speeds as frequency increases. As illustrated in Fig. 7b, regions in the DMN had stronger anti-correlations with the LDAI than with the LVAI. Regions in the LN had stronger correlations with the LDAI than with the LVAI. The RVAI had stronger correlations with the LVAI than with the LDAI. The core phenomenon of seed by frequency interaction was a common pattern that stronger connection attenuates faster along frequency which leading to comparable FC strength between these networks and the two seeds at slow 2 and slow 1 frequency bands (see also Fig. 3). Furthermore, the strongest FCs appeared at slow 4 and slow 5 in the SN and other networks, respectively. This network-specific frequency effect was another source of seed by frequency interaction.

### The comparison of frequency boundaries between theory-based method and data-driven approach

**Figure 8:**
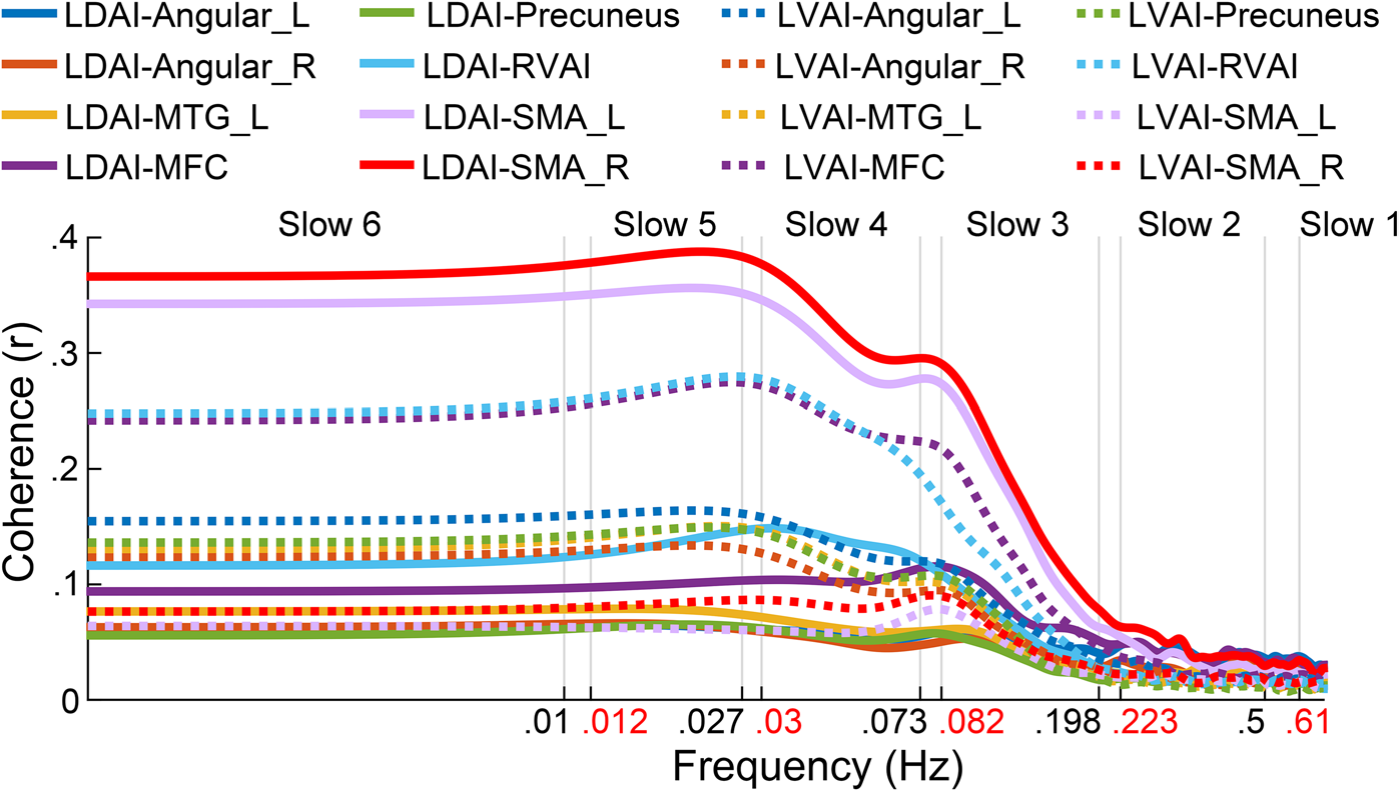
The frequency-dependent coherence between LAI seeds and target regions. The x axis uses the logarithmic coordinate to show the boundaries of six frequency bands used in our work (black) and modified boundaries obtained by the DREAM software (red). The theory-based boundaries fit well with the change of coherence coefficients.

As shown in Fig. 8, the results of coherence analysis showed similar boundary characteristics to the theory-based method. Specifically, the inter-regional relationship was stable between slow 6 and slow 5, dramatically decreased from slow 4 to slow 3, and achieved stability at slow 2 and slow 1. These findings replicated frequency effects in Fig. 6, and suggested that the boundaries defined by the natural logarithm linear law fit well with actual data. In addition, the boundaries we used and those suggested by Gong and colleagues^28^ were both located around the inflection points of coherence curves, indicating the rationality of frequency boundaries used in the current study and suggesting that the natural logarithm linear law applies to the infra-slow frequency range.

## Discussion

As two symmetrical hubs of the SN, the LAI has received much less attention than the RAI. Here we demonstrated seed- and frequency-dependent functional organization of the LAI, which may improve our understanding of the functional integration and functional separation in the LAI and provide a basic framework for future cognitive and clinical investigations of the LAI. Specifically, the LAI was strongly connected to the SN, LN, and DMN in multiple frequency divisions, which is different from its broadband FC pattern and from the FC pattern of RAI. The LDAI had stronger connections with the LN and DMN whereas the LVAI had stronger connections within the SN, showing dissociated FC patterns between the LDAI and LVAI. These dissociated FC patterns had stable and close relationship with the cognitive profiles of LDAI and LVAI. The frequency effect and frequency by seed interaction appeared in most pairs of frequency bands, indicating that frequency and seed interplay with each other to determine the FC pattern of LAI. These main results were reliable and had moderate to large effect sizes.

Both the LDAI and LVAI are linked to the SN, LN, and DMN but with dissociated FC strengths and cognitive profiles, suggesting that the LDAI and LVAI are both functional integrated and functional separated. The functional integration of LDAI and LVAI is largely due to both of them are part of the SN^29^. The LDAI is a dominant region associated with language processing^30,31^ as well as a part of the task positive network^32^ which may account for the correlation with LN and anti-correlation with DMN. Direct fiber projection between LDAI and language regions, the left lateralization of language function, and partial functional separation of LN and frontoparietal network (FPN)^33–36^ may account for strong correlation between the LDAI and LN rather than between LDAI and FPN, further explaining why the LAI is not involved in most attention-demanding tasks and in modulating the interaction between FPN and DMN^6,37,38^. Furthermore, the results of meta-analytic decoding reveal close relationship between the FC maps of LAI and sensory, motor, language, executive, conflict, and DMN related cognitive functions. These functions are in accord with previous findings of the insula function^1,38^, supporting the reliability of FC maps of the LAI.

Beyond functional concordance, there are some systematic deviations for the FCs of LDAI and LVAI. For broadband FC patterns, the LDAI has specific connections with the sensorimotor regions, LN, and DMN whereas the LVAI has specific connections with the rostral ACC and parieto-occipital regions. This dissociation is in line with previous findings^12^. For narrow band FC patterns, the LDAI has stronger connections with LN and DMN whereas the LVAI has stronger connections within SN. The correlation between LVAI and SN mirrors the FC pattern of the RAI^7^. This may be determined by direct fiber connection between symmetric regions of the AI across the corpus callosum^39,40^. An alternative explaination is that the LVAI has more von Economo neurons which project to the ACC forming a basic structure of the SN^29^. Overall, the functional segregation of LDAI and LVAI is supported by their different cytoarchitectures and structural connections^9,29^.

In addition to the structural basis, frequency is an important factor influencing the functional organizations of LDAI and LVAI. Three different FC patterns are observed at broadband, lower frequency (slow 6-slow 3), and higher frequency (slow 2-slow 1) bands. It is suggested that neural activities are composed of scale-free components and multiple oscillations^41,42^. Broadband signal provides valuable information about cross-scale neural activities^12,43^, whereas band-limited information is closely related to neural oscillations^15,17^. The broadband FCs of the LDAI and LVAI are similar to those of the RDAI and RVAI^12,14^, but the broadband FCs of AI in its entirety show hemispheric lateralization^8^. As we hypothesized, this incompatibility with distinctive functions of the LAI and RAI could be resolved with frequency-dependent FC. We observed similar narrow frequency boundaries but different FC patterns of LAI and RAI which may be caused by inter-hemisphere fiber projection via the corpus callosum, given that the same locations of left and right insula rather than different parts of one hemisphere show similar activity patterns^12,39^.

On the other hand, the FC strengths in most regions were weakened from slow 4 to slow 2, forming the transition region of two kinds of narrow band FC patterns and contributing the interaction of seed by frequency. One possibility is that functional integration and functional separation between LAI and RAI and between LAI and other networks need different time scales. This hypothesis has been demonstrated in FC of the RAI^7^ and in top-down and bottom-up coordination at higher frequency range^44^. Alternatively, the spectral fingerprints theory of cognition suggests that one brain region connects with different other ones at different frequency bands to implement different cognitive activities^45,46^. Stable FC patterns of LDAI and LVAI at lower and higher frequency ends may support distinct cognitive functions whereas those at middle frequency range may be an interim between these two states. In addition, regions with significant interaction are the same regions with significant seed effect and frequency effect, indicating that the functional separation of LAI is influenced by frequency. This is different from those effects in the RAI^7^, suggesting the unique FC pattern of LAI.

The frequency-specific FC patterns of LDAI and LVAI are reliable and physiological meaningful which are supported by the following evidence: First, the reliability of current results is guaranteed by stringent data analyses, including long scanning time, noise regression and correction for multiple comparisons. Second, the frequency-specific FC patterns are in line with previous findings such as the broadband FCs of LDAI and LVAI in another independent sample^12^ and similar frequency effect of the RDAI and RVAI^7^. Third, the physiological meaning of high frequency FC was demonstrated by many other studies^19,22,47^. In addition, both LDAI and LVAI have strong connections within the LAI at higher frequency bands which are in line with FC patterns of the RAI and previous findings that higher frequency fluctuations are confined to smaller neural space^7,18,47^. The different FC maps at the same frequency band between LAI and RAI suggest that there is something unique to the LAI, which reflects frequency-dependent FC of the LAI rather than noises because fluctuations of physiological noises would equally affect two hemispheres. Last, these FC patterns are nicely coordinated by their cognitive profiles (see Fig. 5). Some researchers have argued that frequency-dependent inter-regional relationship is determined by particular cognitive processing which is known as the spectral fingerprint of that cognitive processing^45,46,48^. Overall, the functional integration and functional segregation of LDAI and LVAI are frequency-specific.

Our results suggest that combining broadband with narrow band analyses is necessary to comprehensively uncover the functional organization of resting-state neural activities. Some limitations remain. First, the BOLD signal cannot afford a direct link between these FCs and neural activities. These frequency-dependent FC patterns maybe not entirely neural activities because BOLD signal fluctuations contain various non-neuronal physiological processes in different frequency bands, such as ion concentration dynamics^49^, glial cell activities^50^, and respiration and cardiac pulsations^51^. Many recent studies have demonstrated that the head motion^52^, global signal^53^, white matter^54^, cerebrospinal fluid^55^, respiratory^56^, and cardiac signal^57^ contain lots of meaningful physiological and pathological information. Some of these signals even have frequency-specific effects on FC^57,58^. Second, although Buzsáki inferred distinctive neural oscillations from slow 4 to slow 2 according to a comparable frequency interval between successive oscillations in a log axis of coordinates^18^, there is yet no consensus on how many frequency bands should the low frequency neural fluctuations be divided into. Tremendous studies have suggested distinctive characteristics in brain disorders and cognitive tasks in different narrow low frequency bands^23,24,59,60^. The cognitive mechanisms of narrow band low frequency BOLD signal fluctuations, however, are largely undetermined. Third, a direct test of structure–function relationship is necessary for illuminating the principle of the functional organization of LAI although multiple frequency neural oscillations dramatically expand the space of inter-regional information communication in a limited anatomical relationship^46^. Lastly, the frequency-dependent functional organization of LAI may alter under various brain states, considering that inter-regional relationship and energy distribution in the low frequency range are altered under different brain states^59,61^. Therefore, the current results cannot be generalized to different task states involving distinctive cognitive processes.

In summary, the frequency-dependent FC patterns of the LDAI and LVAI support multiple neural oscillations in the low frequency range, arguing the essential role of band-limited method in clarifying the rule of low frequency neural activities. The frequency-dependent functional organization of the LAI may serve as a basis for further understanding of the role of LAI in various tasks and pathological situations.

## Methods

### Data acquisition

The resting-state functional magnetic resonance imaging (rfMRI) data of 82 subjects (age range: 22–35; 45 females) were recruited in this study. The sample was selected from the HCP 100 unrelated datasets (https://db.humanconnectome.org) with the criteria that respiratory, cardiac, and head movement data are available in all runs (rfMRI_REST1_LR, rfMRI_REST1_RL, rfMRI_REST2_LR, and rfMRI_REST2_RL). Eighteen subjects lacking these data were excluded. Remaining subjects were the same as those in the previous study^7^, facilitating the comparison between functional organizations of the LAI and RAI. The HCP scanning protocol was approved by the local Institutional Review Board at Washington University in St. Louis. All methods were carried out in accordance with relevant guidelines and regulations. Informed consent was obtained from all subjects. All participants were scanned on a customized Siemens 3-T connectome-Skyra scanner. The imaging parameters used to collect the rfMRI data were as follows: TR = 720 ms; TE = 33.1 ms; flip angle = 52°; 2 mm isotropic voxels (FOV = 208 × 180 mm; 72 slices); multiband factor = 8; echo spacing = 0.58 ms; bandwidth (BW) = 2290 Hz/px; volumes = 1200. Full details on the HCP datasets have been described by Van Essen and colleagues^62^.

### Data preprocessing

The data with minimal preprocessing pipeline^63^ was used. This pipeline includes artifact removal, motion correction, and registration to standard space. Standard preprocessing procedure reported by Finn and colleagues was applied to the present study^60^. The procedure included removal of the linear trend, removal of linear components related to the six motion parameters and their first derivatives^60^, regression of respiratory and cardiac noises (down sampled to 1.39 Hz (1/0.72 s) before the regression calculation)^7,60^, regression of the mean time courses of the white matter and cerebrospinal fluid as well as the global signal, smoothing with a full-width half-maximum (FWHM) of 6 mm. Motion parameters and physiological noises were extracted from the documents Movement_Regressors_dt.txt, rfMRI_REST1_LR_Physio_log.txt, rfMRI_REST1_RL_Physio_log.txt, rfMRI_REST2_LR_Physio_log.txt, and rfMRI_REST2_RL_Physio_log.txt, respectively. These procedures were conducted with MathWorks 8.3 and the Data Processing Assistant for Resting-state fMRI (DPARSF 2.3, https://www.restfmri.net/forum/DPARSF)^64^ and were consistent with the previous study about frequency-dependent FC of the RAI^7^. The time series was further band-pass filtered (including six frequency bands: slow 6: 0.001–0.01 Hz, slow 5: 0.01–0.027 Hz, slow 4: 0.027–0.073 Hz, slow 3: 0.073–0.198 Hz, show 2: 0.198–0.5 Hz, and slow 1: 0.5–0.694 Hz). The higher frequency boundary was determined by the Nyquist theorem ((1 / 0.72 s) / 2 ≈ 0.694 Hz), whereas the lower boundary was determined by the length of data (1 / (1200 * 0.72 s) ≈ 0.001 Hz). We also performed high pass filtering (3 cycles per series cutoff or greater than 0.0035 Hz) to test whether the broadband FC of the LDAI and LVAI in the previous study^12^ could be replicated. The filtered time series of each run was normalized by subtracting the mean and dividing by the standard deviation. The normalized time courses were then linked together^7^.

There are several problems that need clarification. First, the global signal was regressed out to improve the specificity of correlations for different seeds as suggested by Murphy and Fox^65^. Considering that it has no significant influence on FC maps of the RAI^7^, we suggest that global signal regression should have no impact on FC maps of the LAI the symmetrical structure of the RAI. Second, the frequency division approach used here was an extension of frequency division of high frequency neural oscillations to the low frequency range^18^. It was one of the most used methods for dividing low frequency blood oxygen level dependent (BOLD) fluctuations^20,23,42,66,67^, facilitating the comparison of different research results. It is worth noting that there is no consensus on how to divide low frequency (< 1 Hz) BOLD signals into narrow frequency bands because the physiological and psychological significances of BOLD signal are largely undetermined^18,68–70^. Therefore, a considerable amount of work needs to be done before a widely accepted frequency division approach is established. For instance, based on the natural logarithm linear law, a recent study proposed more precision frequency boundaries according to the sampling period and number of samples in different studies^58^. Advantages like this may provide more precision description for the psychological and physiological meanings of low frequency neural oscillations. In the current study, we adopted the coherence analysis, a data-driven approach, to verify the rationality of these theory-based frequency boundaries, reconciling the natural logarithm linear law with actual data.

### FC analysis and statistics

To investigate FC patterns of the LDAI and LVAI and their frequency effects, the whole brain FC was conducted based on the LDAI (mean coordinate: x = −38, y = 6, z = 2) and LVAI (mean coordinate: x = −33, y = 13, z = −7) seeds defined by Deen and colleagues^12^ in aforementioned broadband and narrow frequency bands. Voxel-wise temporal correlation was computed and transformed to Fisher's z value. The FC maps were defined using one-sample T-test. FCs were compared between two seeds and between any pair of frequency bands using paired-samples T-test. Interaction between seed and frequency band was evaluated with the repeated measures analysis of variance (ANOVA). These statistics were conducted using SPM12 software (www.fil.ion.ucl.ac.uk/spm). Each resulting map was corrected using the family-wise error (FWE) method (*p* < 0.05/the number of voxel within the statistical map) for multiple comparisons^71,72^. Cohen’s d and Cohen’s f were computed as indices of effect size of T-test and F-test^73^, respectively.

Of note, the low to moderate reliability of FC has been stressed in recent studies which seriously impedes its research and clinical application^74,75^. In this study, three methods were used to improve the reliability of FC results. First, four runs were linked together to increase the length of data. The length of data was 57.6 min for each subject which ensures moderate to good reliability for resting-state FC analysis^76^. Second, the family-wise error method was used to eliminate false positive results in a relative large sample (N = 82). Third, motion parameters, physiological noises, linear trend, white matter, cerebrospinal fluid, and the global signals were regressed out to avoid possible influences from noises. Furthermore, the results of broadband FCs of LDAI and LVAI mirrored findings in another independent sample^12^ and the frequency characteristics were similar but the FC maps were different for LAI and RAI^7^. These operations and evidence indicate the reliable and unique FC pattern of the LAI.

### Meta-analytic decoding for the cognitive correlations of FC maps

To decode the potential psychological processes associated with each frequency-specific LAI FC map, we examined the FC pattern of each frequency band using NeuroSynth (https://www.neurosynth.org/)^77^. Guided by previous works in the meta-analytic decoding of FC maps 78,79, we first converted the t maps obtained in one-sample T-test into z maps and then submitted them to the Neurosynth system for meta-analytic decoding. Next, psychological processes with mean correlation coefficient of six frequency bands greater than 0.1 or lower than −0.1 were retained. Only the item with the strongest correlation among all related items (e.g., episodic, episodic memory) was retained to avoid reduplicative selection. Line charts were plotted for the resulting 17 distinct terms that represent the potential psychological processes of each FC map.

### Verification of frequency boundaries

The coherence analysis was adopted to verify the rationality of the theory-based frequency boundaries used here. The coherence analysis, as a data-driven method, was often used to describe the frequency characteristics of functional connectivity without the requirement of band-limited filtering^21,80^. We first extracted the signals in 2 LAI seeds and 8 target regions with significant interaction of frequency by seed (see Fig. 7) from the unfiltered data. The between-signal coherence was then calculated by employing the function ‘mscohere’ in MATLAB 8.4, the square root of the raw value was defined as the coherence coefficient (r). The r curves were demarcated by theory-based frequency boundaries.

## Data Availability

The MRI datasets used in this study are available to the public from the Human Connectome Project (100 unrelated release; https://db.humanconnectome.org).
